# Seltene Erkrankungen und Digitalisierung im Kontext des Nationalen Aktionsbündnisses für Menschen mit Seltenen Erkrankungen (NAMSE)

**DOI:** 10.1007/s00103-022-03597-w

**Published:** 2022-10-14

**Authors:** Theda Wessel, Katharina Heuing, Miriam Schlangen, Birgit Schnieders, Markus Algermissen

**Affiliations:** 1 Bundesministerium für Gesundheit, Friedrichstr. 108, 10117 Berlin, Deutschland; 2 NAMSE Geschäftsstelle, Bonn, Deutschland

**Keywords:** Seltene Erkrankungen, Digitalisierung, Nationales Aktionsbündnis für Menschen mit Seltenen Erkrankungen (NAMSE), Elektronische Patientenakte (ePA), Europäischer Gesundheitsdatenraum (European Health Data Space – EHDS), Rare diseases, Digitization, National Action League for People with Rare Diseases (NAMSE), Electronic patient record (ePA), European Health Data Space (EHDS)

## Abstract

Menschen mit Seltenen Erkrankungen stehen im Gesundheitssystem vor besonderen Herausforderungen. Die Seltenheit der einzelnen Erkrankungen erschwert aus strukturellen, medizinischen und ökonomischen Gründen sowohl die medizinische Versorgung als auch die Forschung zu den entsprechenden Themen. Im Jahr 2010 wurde das Nationale Aktionsbündnis für Menschen mit Seltenen Erkrankungen (NAMSE) auf Initiative des Bundesministeriums für Gesundheit gemeinsam mit dem Bundesministerium für Bildung und Forschung und der Allianz Chronischer Seltener Erkrankungen sowie 25 weiteren Bündnispartnern ins Leben gerufen. Das NAMSE ist seitdem die zentrale Koordinierungs- und Kommunikationsplattform für Menschen mit Seltenen Erkrankungen in Deutschland und hat das Ziel, die gesundheitliche Situation der Betroffenen zu verbessern.

Wesentliche im NAMSE konsentierte Bedarfe an die Digitalisierung im Gesundheitswesen betreffen insbesondere die Vernetzung von Versorgungseinrichtungen, die Generierung von Wissen für die Forschung und die Verbreitung von Informationen. Zielsetzung ist dabei ein gemeinsamer und sicherer Datenraum mit interoperablen Praxis- und Klinikinformationssystemen und einheitlichen semantischen Standards. Der präzisen Kodierung von Seltenen Erkrankungen kommt dabei eine besondere Bedeutung zu.

In den kommenden Jahren müssen wichtige bereits angestoßene Prozesse im Sinne der Betroffenen gestaltet und begleitet werden. Dazu gehören unter anderem die deutsche Genominitiative genomDE, die elektronische Patientenakte und die Vernetzung im europäischen Raum. Damit die vielfältigen Initiativen und Projekte ineinandergreifen können, bedarf es klarer Zielsetzungen im Rahmen eines digitalen Gesamtkonzeptes, für welches das NAMSE wichtige Beiträge leistet.

## Abstract (en)

People with rare diseases face specific challenges within the healthcare system. Due to the rarity of the individual diseases, both medical care and research are made difficult for structural, medical, and economic reasons. In 2010, the National Action League for People with Rare Diseases (NAMSE) was founded by the German Federal Ministry of Health, the German Federal Ministry of Education and Research, the Alliance for Chronic Rare Diseases, as well as 25 other partners. Since then, NAMSE has been the central coordination and communications platform for people with rare diseases in Germany and aims to improve the health and quality of life of those affected.

As part of the consensus process, NAMSE has formulated requirements regarding digitization in the German healthcare system. These requirements aim towards connecting healthcare institutions, generating knowledge for research purposes, and improving the flow of information. The main objective is a collective and secure health data space with interoperable clinic information systems and uniform semantic standards. The precise coding of rare diseases is of particular importance.

In the coming years, important processes that have already been initiated must be designed and supported in the interest of people with rare diseases. These include the German genome initiative genomDE, the implementation of the electronic patient record, and activities towards a European Health Data Space. In order for the diverse initiatives and projects to mesh, clear objectives are required as part of an overall digital concept to which NAMSE makes important contributions.

Es wird geschätzt, dass etwa 4 Mio. Menschen in Deutschland von einer Seltenen Erkrankung betroffen sind [1]. Eine belastbare Zahl liegt für Deutschland nicht vor. Dies ist nur eine von vielen Fragestellungen im Bereich Seltene Erkrankungen, die durch Fortschritte in der Digitalisierung im Gesundheitswesen beantwortet werden können. Im Folgenden werden die Ziele und Arbeitsweise des Nationalen Aktionsbündnisses für Menschen mit Seltenen Erkrankungen (NAMSE) vorgestellt. Dabei liegt der Fokus auf der Bedeutung der Digitalisierung als Beitrag zur Verbesserung der gesundheitlichen Situation von Menschen mit Seltenen Erkrankungen.

## Das Nationale Aktionsbündnis für Menschen mit Seltenen Erkrankungen (NAMSE)

Menschen mit Seltenen Erkrankungen stehen vor besonderen Herausforderungen – auch und gerade im Gesundheitswesen. Mehr als 70 % der über 6000 Seltenen Erkrankungen sind genetisch bedingt [2]. Oft verlaufen sie chronisch und gehen mit einer eingeschränkten Lebensqualität und -erwartung einher. Die Seltenheit der einzelnen Erkrankungen erschwert aus strukturellen, medizinischen und ökonomischen Gründen sowohl die medizinische Versorgung der Betroffenen als auch die Forschung zur Verbesserung von Diagnose und Therapie. Vor diesem Hintergrund wurden im Jahr 2009 sowohl die „Empfehlung des Rates [der Europäischen Union …] für eine Maßnahme im Bereich seltener Krankheiten“ [3] als auch die Ergebnisse einer vom Bundesministerium für Gesundheit (BMG) in Auftrag gegebenen Studie „Maßnahmen zur Verbesserung der gesundheitlichen Situation von Menschen mit seltenen Erkrankungen in Deutschland“ [4] veröffentlicht. In der Studie wurden die Versorgungssituation von Menschen mit Seltenen Erkrankungen in Deutschland dargestellt, prioritäre Handlungsfelder abgeleitet sowie Verbesserungsvorschläge entwickelt.

### Gründung des NAMSE

Das NAMSE wurde im Jahr 2010 auf gemeinsame Initiative des BMG, des Bundesministeriums für Bildung und Forschung (BMBF) und der Allianz Chronischer Seltener Erkrankungen (ACHSE e. V.) sowie 25 weiterer Bündnispartner – ausschließlich Spitzen- und Dachverbände der wesentlichen Akteure im Gesundheitswesen – ins Leben gerufen (Infobox 1). Das NAMSE ist seitdem die zentrale Koordinierungs- und Kommunikationsplattform für Menschen mit Seltenen Erkrankungen mit dem Ziel, die gesundheitliche Situation der Betroffenen zu verbessern.

### Der Nationale Aktionsplan für Menschen mit Seltenen Erkrankungen

Im Jahr 2013 wurde der im Rahmen des NAMSE konsentierte Nationale Aktionsplan für Menschen mit Seltenen Erkrankungen mit 52 Maßnahmenvorschlägen veröffentlicht. Mit dem vom BMG finanzierten Projekt WB-NAPSE wurde die Umsetzung des Nationalen Aktionsplans von 2015–2017 wissenschaftlich begleitet [5]. Darüber hinaus wurde der Umsetzungsstand durch Monitoring- und Statusberichte der NAMSE-Geschäftsstelle dokumentiert [6]. Für 2022 hat das BMG erneut ein Gutachten in Auftrag gegeben, welches nach 2009 die Versorgungssituation von Menschen mit Seltenen Erkrankungen in Deutschland analysieren und prioritäre Handlungsfelder ableiten soll.

### Wichtige Entwicklungen der letzten Jahre

Seit der Veröffentlichung des Nationalen Aktionsplans sind auf Initiative des NAMSE unter anderem folgende wichtige Entwicklungen zur Verbesserung der gesundheitlichen Situation von Menschen mit Seltenen Erkrankungen angestoßen worden [7]:

- Auf der Grundlage des mit dem Gesetz zur Stärkung des Pflegepersonals (Pflegepersonal-Stärkungsgesetz, PpSG) eingeführten § 136c Absatz 5 Fünftes Buch Sozialgesetzbuch (SGB V) wurden durch den Gemeinsamen Bundesausschuss (G-BA) Ende 2019 erstmals die besonderen Aufgaben von Zentren für Seltene Erkrankungen definiert sowie bundeseinheitliche Qualitätsanforderungen festgelegt [8]. Ausgangspunkt für die Ausgestaltung waren die vom NAMSE entwickelten und konsentierten Anforderungskataloge [9].
- 2021 wurde unter Einbindung einer unabhängigen Zertifizierungsstelle ein Zertifizierungsverfahren für Zentren für Seltene Erkrankungen auf den Weg gebracht.
- Orphanet Deutschland, das als nationaler Partner des europäischen Referenzportals zu Seltenen Erkrankungen und Orphan Drugs unter anderem die Orphanet-Nomenklatur ins Deutsche übersetzt, konnte Anfang 2021 an das Bundesinstitut für Arzneimittel und Medizinprodukte (BfArM) angegliedert und damit verstetigt werden.
- In der Abteilung Kodiersysteme des BfArM wurde über das Patientendaten-Schutz-Gesetz (PDSG) Anfang 2021 eine Stelle für Seltene Erkrankungen etabliert, wodurch BMG-geförderte Projekte zur Kodierung Seltener Erkrankungen verstetigt wurden, um internationale Klassifikationen zu Seltenen Erkrankungen mit den gesetzlichen Diagnoseklassifikationen und Terminologien zu verknüpfen.
- Mit dem Gesetz zur Stärkung der Versorgung in der gesetzlichen Krankenversicherung (GKV-Versorgungsstärkungsgesetz) wurde der Innovationsfonds beim G‑BA eingerichtet und mit dem Digitale-Versorgung-Gesetz (DVG) bis Ende 2024 verlängert. Im Rahmen des Innovationsfonds wurden bzw. werden verschiedene Projekte von hoher Bedeutung für Seltene Erkrankungen gefördert, wie beispielsweise TRANSLATE-NAMSE (Verbesserung der Versorgung von Menschen mit seltenen Erkrankungen) und ZSE-DUO (Duale Lotsenstruktur zur Abklärung unklarer Diagnosen in Zentren für Seltene Erkrankungen), sowie Projekte zur Leitlinienentwicklung [10].

Gleichzeitig sind in den letzten Jahren die Europäischen Referenznetzwerke (ERN) entstanden, an denen rund 200 deutsche Versorgungseinrichtungen aktiv beteiligt sind.

### Arbeitsweise des NAMSE

**Figure 1:**
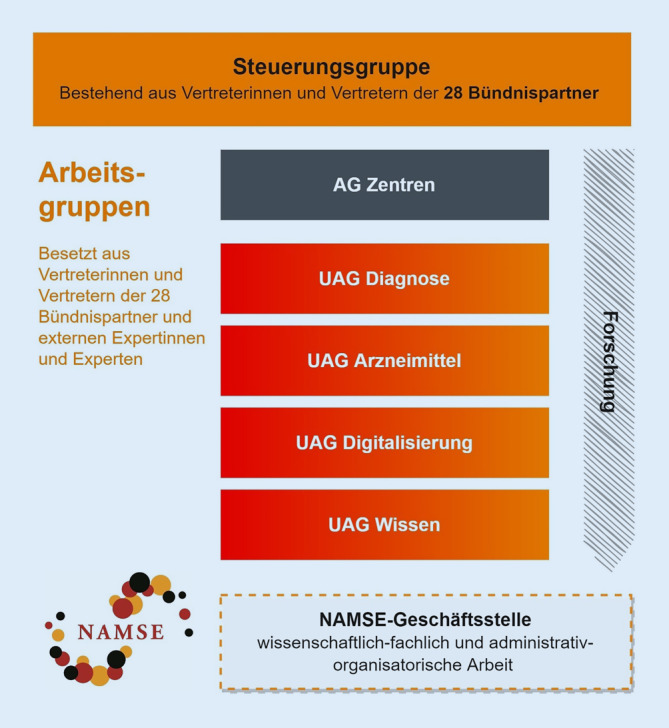

Die Arbeit des NAMSE wird seit seiner Gründung durch eine Geschäftsstelle koordiniert. Die Steuerungsgruppe, bestehend aus den Bündnispartnern, tagt 2‑ bis 3‑mal pro Jahr. Darüber hinaus werden nach Bedarf Arbeits- und Unterarbeitsgruppen eingerichtet, die mehrfach im Jahr tagen und im engen Austausch mit Expertinnen und Experten bestimmte Themenfelder bearbeiten, wie beispielsweise Digitalisierung (Abb. 1). Dabei werden nach Möglichkeit alle relevanten Entwicklungen im deutschen Gesundheitssystem beobachtet und auf ihre Bedeutung für die Seltenen Erkrankungen hin analysiert. Gleichzeitig werden spezifische Ziele formuliert, um der besonderen Situation von Menschen mit Seltenen Erkrankungen gerecht zu werden. Das NAMSE als zentrale Koordinierungs- und Kommunikationsplattform ist dabei für ein gemeinsames und zielorientiertes Handeln von hoher Bedeutung. Die erreichten Fortschritte beruhen darüber hinaus in beträchtlichem Maße auf dem persönlichen Einsatz vieler einzelner Akteure, die sich mit Ausdauer, Ideenreichtum und Gestaltungskraft in ihren Institutionen, in den Zentren, im Rahmen von Stiftungen und in der Selbsthilfe für die Belange von Menschen mit Seltenen Erkrankungen einsetzen.

## Digitalisierung und Seltene Erkrankungen

Seltene Erkrankungen zeichnen sich oftmals dadurch aus, dass nur wenige Expertinnen und Experten zur Verfügung stehen, verschiedene medizinische Fachrichtungen in Bezug auf Diagnose und Therapie zusammenarbeiten müssen und die jeweilige Erkrankung wenig beforscht ist. Auch die Selbsthilfe muss sich häufig über große räumliche Distanz organisieren. Die Digitalisierung bietet daher insbesondere im Hinblick auf *Vernetzungsaspekte*, die *Generierung von Wissen* und die *Verbreitung von Informationen* eine große Chance für Menschen mit Seltenen Erkrankungen. Vor diesem Hintergrund hat das NAMSE vordringliche Bedarfe an die Digitalisierung konsentiert [11], von denen einige prioritäre im Folgenden dargestellt werden.

### Vernetzung in der Versorgung

Menschen mit Seltenen Erkrankungen müssen für eine spezialisierte medizinische Versorgung häufig Grenzen überschreiten: über Sektoren hinweg – also zwischen Praxen und Kliniken –, zwischen verschiedenen Abteilungen innerhalb von Kliniken, zwischen verschiedenen Zentren, über Bundesland- und manchmal auch über Ländergrenzen hinweg. Wenn es an diesen Schnittstellen zu Informationsverlusten kommt, kann dies für Betroffene und Leistungserbringer nicht nur frustrierend sein, sondern auch die Patientensicherheit erheblich beeinträchtigen, z. B. wenn wegen fehlender Vorbefunde notwendige Untersuchungen unterbleiben oder mehrfach vorgenommen werden, relevante Risikofaktoren nicht berücksichtigt werden oder keine bzw. eine falsche Diagnose gestellt wird. Welche Einrichtungen dabei jeweils vernetzt zusammenarbeiten müssen, variiert in Abhängigkeit von Krankheitsbild, Alter der Betroffenen und deren Wohnort. Daher können sogenannte Insellösungen, die sich auf einzelne Versorgungseinrichtungen, auf bestimmte Sektoren oder geografische Regionen beschränken, im Kontext von Seltenen Erkrankungen auf Dauer nicht zielführend sein. Vielmehr muss ein Gesamtnetzwerk entstehen. Daraus abgeleitet ergibt sich folgender im NAMSE konsentierte Bedarf:

- Informationssysteme in Kliniken und Praxen sollen deutschlandweit interoperabel sein.

### Wissen generierende Versorgung und Forschung

Forschung auf dem Gebiet der Seltenen Erkrankungen ist einerseits von einem rasanten Erkenntnisgewinn gekennzeichnet – wie beispielsweise im Bereich der genomischen Medizin –, andererseits durch große Lücken, so dass oftmals selbst grundlegende Erkenntnisse zum natürlichen Verlauf einer Erkrankung nur bruchstückhaft vorliegen. Damit Betroffene von neuen Erkenntnissen möglichst umgehend profitieren und gleichzeitig die im klinischen Alltag erhobenen Daten auch die Forschung voranbringen können, müssen Versorgung und Forschung Hand in Hand gehen. Für einige Erkrankungen bzw. Erkrankungsgruppen liegen umfangreiche Register vor, über die sich viele klinische Forschungsfragen beantworten lassen [12]. Allerdings können diese aufgrund von uneinheitlichen Datenstrukturen teilweise nur händisch bzw. lediglich halbautomatisiert befüllt werden. Angesichts von mehr als 6000 Seltenen Erkrankungen und begrenzten Ressourcen ist es mit der bisherigen Herangehensweise unrealistisch, für alle Erkrankungsgruppen Einzelregister einzurichten und nachhaltig vorzuhalten. Es erscheint daher unerlässlich, die im klinischen Alltag generierten Daten im Sinne einer *Wissen generierenden Versorgung* auch für Forschungszwecke auswertbar zu machen. Dazu bedarf es zum einen der Vernetzung von Versorgungseinrichtungen in einer gemeinsamen, geschützten Dateninfrastruktur, um auch dezentral gehaltene klinische Datenbestände in digitale Auswertungen mit einbeziehen zu können. Zum anderen müssen durch eine gemeinsame semantische Strategie einheitliche Datenstrukturen geschaffen werden. Daraus abgeleitet ergeben sich folgende im NAMSE konsentierten Bedarfe:

- Deutschland soll eine gemeinsame semantische Strategie im Gesundheitswesen entwickeln, die die digitale Auswertung von klinischen Befunden ermöglicht.
- Datenstandards für Seltene Erkrankungen, die bereits auf nationaler, europäischer und internationaler Ebene etabliert sind, sollen regelhaft berücksichtigt werden.
- Genomische Untersuchungen sollen immer eine standardisierte Phänotypisierung (Beschreibung der klinischen Befunde) als Voraussetzung haben.

### Kodierung Seltener Erkrankungen

Einen besonderen Stellenwert im Rahmen einer einheitlichen semantischen Strategie hat die genaue Benennung der Diagnose, von der alle anderen digitalen Anwendungen in hohem Maße abhängen. Zu Abrechnungszwecken werden Diagnosen in Deutschland mithilfe der ICD-10-GM verschlüsselt, also der deutschen Version der Internationalen Klassifikation der Krankheiten. Darin ist jedoch nur ein Bruchteil der Seltenen Erkrankungen eindeutig benannt. Im Rahmen von zwei durch das BMG geförderten Projekten wurde die Datei Alpha-ID-SE entwickelt, in der die internationalen Orpha-Kennnummern zu Seltenen Erkrankungen und ICD-10-GM-Codes über eine Alpha-ID miteinander verknüpft sind. Damit wird eine vereinfachte, einheitliche und standardisierte Kodierung der Seltenen Erkrankungen in Deutschland ermöglicht. Die Datei Alpha-ID-SE wird – auch auf der Grundlage von Hinweisen aus den Versorgungseinrichtungen – immer wieder durch neue Einträge und weitere deutsche Synonyme aktualisiert und ergänzt. Erweiterungsvorschläge können über klassi@bfarm.de an das BfArM gerichtet werden (*Siehe auch Beitrag von Hebestreit in diesem Themenheft*). Es ist geplant, mit der Bekanntmachung des Zeitpunkts der Inkraftsetzung der ICD-10-GM im Bundesanzeiger nach § 301 Absatz 2 Satz 4 SGB V für die Anwendung der ICD-10-GM für das Jahr 2023 zu bestimmen, dass in der *stationären* Versorgung ab dem Jahr 2023 die Schlüsselnummer der ICD-10-GM und zusätzlich eine Orphanet-Kennnummer anhand der Datei Alpha-ID-SE anzugeben sind, sofern sie für die zu kodierende Erkrankung vorliegen. Nach der entsprechenden Bekanntmachung ist die Orphanet-Kennnummer Teil der bei Diagnoseangaben zu übermittelnden Informationen. Für den *ambulanten* Bereich steht die Datei Alpha-ID-SE zur freiwilligen Nutzung kostenlos beim BfArM zur Verfügung.

Die genaue, digital auswertbare Benennung der Diagnose ist nicht nur für *Abrechnungszwecke* relevant, sondern insbesondere auch für *Epidemiologie, Forschung* und die Nutzung von* künstlicher Intelligenz (KI)*. Sie ist darüber hinaus von elementarer Bedeutung für die *Patientensicherheit*, damit im klinischen Kontext – auch und gerade im Notfall – besondere Risikofaktoren und therapeutische Notwendigkeiten umgehend berücksichtigt werden können. Daraus abgeleitet ergibt sich folgender im NAMSE konsentierte Bedarf:

- Die präzise Kodierung von Seltenen Erkrankungen soll auf der Grundlage von internationalen Klassifikationen deutschlandweit einheitlich und verbindlich sein.

### Datenschutz und Einwilligung der Betroffenen

Menschen mit Seltenen Erkrankungen haben meist eine besonders hohe Bereitschaft, ihre im Rahmen der Versorgung erhobenen Daten für die medizinische Forschung zur Verfügung zu stellen [13]. Gleichzeitig ist vielen Betroffenen sehr bewusst, dass es sich dabei um besonders sensible Daten handelt, insbesondere bei genetischen Befunden. Vor diesem Hintergrund besteht ein hohes Interesse daran, dass die Datennutzung für die Betroffenen steuerbar ist und keine unkontrollierte Weitergabe an Dritte erfolgt. Daraus abgeleitet ergeben sich folgende im NAMSE konsentierten Bedarfe:

- Gesundheitsdaten dürfen einen geschützten Datenraum nicht verlassen.
- Die Rahmenbedingungen für die Auswertung von Gesundheitsdaten von Menschen mit Seltenen Erkrankungen sollen unter Einbeziehung von Patientenverbänden im Rahmen eines Gesamtkonzeptes zur Sekundärnutzung von Gesundheitsdaten erarbeitet werden.
- Es sollen praktikable und national einheitliche Lösungen für die Aufklärung und Einholung der Einwilligung von Patientinnen und Patienten in Datenspeicherung und -nutzung gefunden werden, die eine handhabbare und effiziente Anwendung für Leistungserbringer im klinischen Alltag ermöglichen. Es sollen zudem Modelle entwickelt werden, die es Betroffenen erlauben, möglichst transparent sehen und steuern zu können, zu welchem Zweck sie welche Daten zur Verfügung stellen.

### Information und Kommunikation über räumliche Distanz

Seltene Erkrankungen erfordern im besonderen Maße effiziente Informations- und Kommunikationsmöglichkeiten über räumliche Distanz. Dies gilt für Betroffene, Versorgende und Forschende gleichermaßen. Die Kommunikation unterstützen beispielsweise telemedizinische Angebote sowie die kürzlich für Deutschland entwickelte Plattform *KONSIL-SE *[14], die analog zum europäischen Clinical Patient Management System (CPMS) standortübergreifende Fallkonferenzen ermöglicht. Im Hinblick auf die Suche nach spezialisierten Versorgungseinrichtungen, geeigneten Studien oder Registern, Ansprechpartnerinnen und -partnern in der Selbsthilfe und Informationen zu Krankheiten seien an dieser Stelle folgende digitale Informationsangebote zu Seltenen Erkrankungen genannt:

- **se-atlas**: Atlas spezialisierter Versorgungseinrichtungen für Seltene Erkrankungen mit ihren Versorgungsangeboten und Patientenorganisationen [15],
- **Orphanet Deutschland**: Nationale Plattform des europäischen Referenzportals für Seltene Erkrankungen und Orphan Drugs am BfArM [16].

### Weitere digitale Anwendungen und Projekte des BMG

Über die genannten Aspekte hinaus bietet die Digitalisierung weitere Möglichkeiten, z. B. den Einsatz von KI bei der Diagnosestellung mithilfe von Algorithmen oder Gesichtserkennung (siehe auch Beitrag von Krawitz in diesem Themenheft). Auch das BMG fördert Projekte in diesem Bereich. Eine Übersicht der BMG-geförderten Projekte mit Bezug zu Digitalisierung und Seltenen Erkrankungen ist in Infobox 2 dargestellt.

### Forschungsförderung durch das BMBF

Das BMBF fördert seit 2003 nationale Verbünde, die die Ursachen Seltener Erkrankungen erforschen und neue Diagnosemöglichkeiten und Therapieansätze entwickeln. Für diese Forschung wurden bislang über 108 Mio. € bereitgestellt. Ziel der Maßnahme ist es, die bestehenden Kompetenzen in der anwendungsorientierten Grundlagenforschung, der klinischen Forschung und der Versorgungsforschung zu Seltenen Erkrankungen zu bündeln. Das BMBF beteiligt sich außerdem an europäischen Förderinitiativen zu Seltenen Erkrankungen, um internationale Forschungsverbünde zu fördern – aktuell im European Joint Programme on Rare Diseases (EJP RD).

Die Nutzung von Patientendaten im Rahmen von Krankheitsregistern spielt in diesen Verbünden eine wichtige Rolle. Die Medizininformatik-Initiative (MII) des BMBF legt dabei entscheidende Grundlagen für die standortübergreifende Erschließung und Nutzung digitalisierter Gesundheitsdaten. 4 Konsortien entwickeln dafür seit dem Jahr 2018 innovative IT-Lösungen, sogenannte Use Cases, für konkrete medizinische Anwendungen und zeigen die Möglichkeiten moderner digitaler Dienstleistungen und Infrastrukturen im Gesundheitsbereich für Forschung und Versorgung anhand von konkreten Anwendungsbeispielen. Alle Universitätsklinika in Deutschland sind beteiligt. Hier werden IT-Infrastrukturen, sogenannte Datenintegrationszentren aufgebaut und vernetzt. In diesen Zentren werden die rechtlichen, technischen und organisatorischen Voraussetzungen geschaffen, um Forschungs- und Versorgungsdaten einheitlich erheben und standortübergreifend verknüpfen zu können. Einer dieser Use Cases ist „Collaboration on Rare Diseases“ (CORD_MI), den das BMBF mit 6 Mio. € fördert (*Siehe auch Beitrag von Schepers in diesem Themenheft*).

### Aktuelle Entwicklungen

In den letzten Jahren sind in Deutschland viele Prozesse angestoßen worden, um die Digitalisierung im deutschen Gesundheitswesen voranzubringen. Damit hat sich der Handlungsrahmen aller Akteure im Gesundheitswesen erheblich erweitert. Hier ist insbesondere die elektronische Patientenakte (ePA) zu nennen, die seit Januar 2021 allen gesetzlich Versicherten über ihre Krankenkassen zur Verfügung steht und in der medizinische Befunde und Informationen aus vorhergehenden Untersuchungen und Behandlungen über Praxis- und Krankenhausgrenzen hinweg gespeichert werden können. Die ePA ist damit das zentrale Instrument der sektorenübergreifenden Zusammenarbeit (*Siehe auch Beitrag von Rashid in diesem Themenheft*). Auch die Nutzung von Telekonsilien ist im Bereich der Seltenen Erkrankungen sektorenübergreifend möglich und kann damit räumliche Distanz überbrücken. Eine weitere wichtige Entwicklung ist die Einführung von digitalen Gesundheitsanwendungen, wie beispielsweise „Apps“. Gerade in diesem Bereich setzt der Rechts- und Vergütungsrahmen besondere Anreize, auch für Seltene Erkrankungen innovative und bedarfsgerechte Angebote zu entwickeln. Den erweiterten Rechtsrahmen gilt es konsequent im Sinne der Betroffenen mit Leben zu füllen und fortzuentwickeln.

Zu einer Wissen generierenden Versorgung mit digitaler Infrastruktur soll auch das Modellvorhaben zur umfassenden Diagnostik und Therapiefindung mittels Genomsequenzierung bei Seltenen und bei onkologischen Erkrankungen beitragen, das im Sommer 2021 in § 64e SGB V aufgenommen wurde. Im Rahmen eines strukturierten klinischen Behandlungspfads und einer darauf aufbauenden Zusammenführung von klinischen und genomischen Daten in einer Dateninfrastruktur soll die Analyse der gewonnenen Daten zur Verbesserung der medizinischen Versorgung erleichtert und auch eine Bereitstellung der Daten für die Forschung ermöglicht werden.

Im europäischen Kontext wird derzeit ein Regelungsvorschlag zur Schaffung eines europäischen Gesundheitsdatenraums (*European Health Data Space*, EHDS) erarbeitet. Der EHDS soll einen einheitlichen Rechtsrahmen für die grenzüberschreitende Nutzung von Gesundheitsdaten sowohl im Bereich der Versorgung (Primärnutzung) als auch für Forschung und Innovation (Sekundärnutzung) schaffen. Eine länderübergreifende, datenschutzkonforme Verknüpfung verschiedener Datenbestände kann insbesondere für die Forschung zu Seltenen Erkrankungen neue Impulse geben und dazu beitragen, den Erkenntnisgewinn weiter zu beschleunigen und bestehende Wissenslücken zu schließen.

## Fazit und Ausblick

Seltene Erkrankungen stellen im Hinblick auf die Digitalisierung keinen exotischen Sonderfall dar, sondern machen wie in einem Brennglas deutlich, wie hilfreich digitale Lösungen im Gesundheitssystem für die Vernetzung von Versorgungseinrichtungen, die Generierung von Wissen für die Forschung sowie die Verbreitung von Informationen sein können. Voraussetzung hierfür ist ein *gemeinsamer und sicherer Datenraum* mit *interoperablen Praxis- und Klinikinformationssystemen* und *einheitlichen semantischen Standards*, in denen Seltene Erkrankungen berücksichtigt sind. Die Bedürfnisse der Betroffenen müssen dabei im Mittelpunkt stehen. In den kommenden Jahren müssen wichtige bereits angestoßene Prozesse gestaltet und begleitet werden. Dazu gehören unter anderem die deutsche Genominitiative genomDE, die ePA und die Vernetzung im europäischen Raum. Damit die vielfältigen Initiativen und Projekte ineinandergreifen können, bedarf es klarer Zielsetzungen im Rahmen eines digitalen Gesamtkonzeptes. Hierzu leistet das NAMSE als Koordinierungs- und Kommunikationsplattform, in der die wesentlichen Akteure des deutschen Gesundheitswesens vernetzt sind, einen wichtigen Beitrag.

### Infobox 1 Bündnispartner des Nationalen Aktionsbündnisses für Menschen mit Seltenen Erkrankungen (NAMSE)

- **ACHSE e.** V. Allianz Chronischer Seltener Erkrankungen*
- Arbeitsgemeinschaft der Obersten Landesgesundheitsbehörden (AOLG)
- Arbeitsgemeinschaft der Wissenschaftlichen Medizinischen Fachgesellschaften e. V. (AWMF)
- Beauftragter der Bundesregierung für die Belange der Patientinnen und Patienten
- Bundesarbeitsgemeinschaft Selbsthilfe von Menschen mit Behinderung und chronischer Erkrankung und ihren Angehörigen e. V. (BAG SELBSTHILFE e. V.)
- Bundesärztekammer (BÄK)
- Bundesministerium für Arbeit und Soziales (BMAS)
- Bundesministerium für Bildung und Forschung (BMBF)*
- Bundesministerium für Familie, Senioren, Frauen und Jugend (BMFSFJ)
- Bundesministerium für Gesundheit (BMG)*
- Bundespsychotherapeutenkammer (BPtK)
- Bundesverband der Pharmazeutischen Industrie e. V. (BPI)
- Bundesverband Medizintechnologie e. V. (BVMed)
- Bundeszahnärztekammer (BZÄK)
- Deutsche Forschungsgemeinschaft (DFG)
- Deutscher Hausärzteverband e. V.
- Deutsche Krankenhausgesellschaft e. V. (DKG)
- Deutscher Pflegerat e. V. (DPR)
- Gemeinsamer Bundesausschuss (G-BA)
- GKV-Spitzenverband
- Kassenärztliche Bundesvereinigung (KBV)
- Kassenzahnärztliche Bundesvereinigung (KZBV)
- Medizinischer Fakultätentag der Bundesrepublik Deutschland e. V. (MFT)
- Orphanet Deutschland
- PKV-Verband der privaten Krankenversicherung e. V.
- Verband der Forschenden Arzneimittelhersteller e. V. (vfa)
- Verband der Universitätsklinika Deutschlands e. V. (VUD)
- Verband der Diagnostica-Industrie e. V. (VDGH)

* Gründungsorganisationen

### Infobox 2 Projekte des Bundesministeriums für Gesundheit (BMG) mit Bezug zu Digitalisierung und Seltenen Erkrankungen

- **genomDE** – Initiative zum Aufbau einer bundesweiten Plattform zur medizinischen Genomsequenzierung
  - https://www.bundesgesundheitsministerium.de/themen/gesundheitswesen/personalisierte-medizin/genomde-de.html
- **OSSE** – Open-Source-Registersystem für Seltene Erkrankungen
  - www.osse-register.de
- **GenKI** – Genetische Beratung zwischen KI und persönlicher Entscheidung
  - https://www.bundesgesundheitsministerium.de/ministerium/ressortforschung-1/handlungsfelder/forschungsschwerpunkte/digitale-innovation/modul-4-smarte-kommunikation/genki.html#:~:text=Ziel%20des%20GenKI%20Projektes%20ist,Vorabinformation%20von%20Patientinnen%20und%20Patienten
- **Leuko-Expert** – KI-basierte Diagnoseunterstützung bei Seltenen Erkrankungen am Beispiel der Seltenen Erkrankung Leukodystrophie
  - https://www.bundesgesundheitsministerium.de/ministerium/ressortforschung-1/handlungsfelder/forschungsschwerpunkte/digitale-innovation/modul-3-smarte-algorithmen-und-expertensysteme/leuko-expert.html
- **TPI – Digitale Biomarker** – Künstliche Intelligenz in der Präzisionsonkologie TPI
  - https://www.bundesgesundheitsministerium.de/ministerium/ressortforschung-1/handlungsfelder/forschungsschwerpunkte/digitale-innovation/modul-2-smarte-datennutzung/tpi.html
- **SATURN** – Smartes Arztportal für Betroffene mit unklarer Erkrankung
  - https://www.uniklinikum-dresden.de/de/das-klinikum/universitaetscentren/zentrum-fuer-medizinische-informatik/bmg-projekt-smartes-arztportal-fuer-betroffene-mit-unklarer-erkrankung-saturn-erfolgreich-gestartet
- **knw Elterndatenbank** – Entwicklung einer digitalen, interaktiven Anwendung als Vernetzungsangebot für Gleichbetroffene (PeerNetApp)
  - https://www.bundesgesundheitsministerium.de/ministerium/ressortforschung-1/handlungsfelder/forschungsschwerpunkte/staerkung-der-gesundheitskompetenz/peernetapp.html
- **ESE-Best** – Evaluation von Schnittstellenmanagementkonzepten bei Seltenen Erkrankungen:
  - Systematische Bestandsaufnahme & Erstellung von Best-Practice-Empfehlungen (Abschlussbericht wird bis November 2022 veröffentlicht sein)
